# The Burden Mental Research Trust: Its Present and Future

**Published:** 1937

**Authors:** R. J. A. Berry

**Affiliations:** Chairman of the Trust and Director of Medical Services, Stoke Park Colony


### THE BURDEN MENTAL RESEARCH TRUST: ITS PRESENT AND FUTURE.

BY

R. J. A. Berry, M.D., F.R.S.E.,

*Chairman of the Trust and*
*Director of Medical Services, Stoke Park Colony.*

---

In January, 1933, this *Journal* recorded the foundation of the Burden Mental Research Trust by Mrs. R. G. Burden, Warden of the National Institutions for Persons requiring Care and Control. Mrs. Burden donated the sum of £10,000, and with the gift expressed her desire that it should primarily be devoted to problems underlying the causation and inheritance of normal and abnormal mentality, and that both principal and interest should be expended over a period of not less than five years. The Burden Mental Research Trust thus came into being, and is administered by a responsible Committee of Management upon which the following bodies are officially represented :—

The British Medical Association.
The Medical Research Council.
The Board of Control.
The Board of Education.
The Galton Eugenics Laboratory of the University of London.
The Royal Medico-Psychological Association.
The Central Association for Mental Welfare.

These representatives, together with Mrs. Burden, the Director of Medical Services at Stoke Park, and Dr. W. Rees Thomas as a co-opted member, form the committee, which meets periodically in offices provided by the British Medical Association, with Dr. Anderson as Secretary.

In 1933 the Committee of Administration made the following major appointments :—

Principal Investigator : J. A. Fraser Roberts, M.A., M.B., Ch.B., D.Sc., F.R.S.E.

Part-time Assistant : R. M. Norman, M.D., D.P.M.

Psychological Assistant : Ruth Griffiths, M.A., Ph.D.

and later made other minor appointments, one of the most important of which was the part-time appointment of a social worker.

The period of five years originally contemplated is now drawing to a close, and it is with great satisfaction that it can now be announced that the results of the work have been deemed so satisfactory that arrangements for the activities of the Trust to be continued indefinitely as a part of the regular work of Stoke Park Colony are under consideration.

From the outset of his researches the Principal Investigator, Dr. Fraser Roberts, has, with the full knowledge and approval of his Committee, taken a wide view of the terms of his reference, and has endeavoured to make the work of the Burden Mental Research complementary to, rather than a mere duplication of, the work of the Darwin Trust. He has also been authorized to consult, at the Trust's expense, such authorities as he from time to time has thought desirable. For their services in this capacity thanks are due to Professor R. A. Fisher, the late Dr. Shepherd Dawson, and Dr. J. G. Greenfield of the Queen's Square National Hospital for Nervous Diseases, all of whom have visited Stoke Park, seen the actual work planned and in operation, and upon which they have advised and reported.

Bearing in mind the objectives of the Burden Trust, namely an enquiry into the causation and inheritance of mentality whether normal or abnormal, work has been carried out along three main lines with a convergent objective :—

1. A complete ascertainment of the mentality of a cross section of a reputedly mentally normal school population.

2. An analogous study of a known and certified mentally abnormal population.

3. An examination after death of the brains of individuals drawn from the classes just mentioned, with a view to ascertaining wherein, how, and why the brains of the mentally abnormal differ from those of the mentally normal.

As regards the *first* of these lines of investigation Dr. Fraser Roberts has undertaken an ascertainment of a very complete cross-section of the population. Much work has been carried out on this population and upon special portions of it, the general aim being an analysis of as many factors as possible that might be considered to have an influence on the development of mentality. Special attention has been paid to problems of backwardness and mental deficiency. The city of Bath having promised the most generous co-operation, that most suitable area was selected, and it was decided to ascertain and give a mental test to all the children whose homes were within the city boundaries on 27th July, 1934, and whose dates of birth fell between 1st September, 1921, and 31st August, 1925, inclusive. The private schools also co-operated most willingly, and apart from a small fraction attending schools remote from Bath the ascertainment and mental testing have been made almost entirely complete. The total number of children included was 3,400. Numerous studies have been and are being made on the entire group, *e.g.* the distribution of intelligence test scores, the resemblance between brothers and sisters, the relation of intelligence to family size, and, for the great majority of the children, an analysis of mentality in relation to health, maternal age at the time of birth, and several other factors.

Important observations are being made on special sections of this child population. The lowest 8 per cent. of Binet Intelligence Quotients form a special group of 260. A median group of 4 per cent. and a top group of 4 per cent. furnish controls. It will be realized that the value for research purposes of a group that is known to be the lowest 8 per cent. of Binet test results for a complete population is very great. The conclusions will help to interpret the findings in the case of other studies that start with institutional defectives.

For the *second* main objective of the Trust there is a wealth of material at Stoke Park Colony. Even before the Burden Trust came into being the most careful records were being made of the physical, mental, and social conditions of mental defectives from many different parts of England and Wales. Since the inception of the Trust this work has been continued and as it has included every new admission there are now available upwards of 1,000 additional consecutive cases. The results to be obtained from these data will not only afford a useful comparison with the normal school population of the previous paragraph, but will furnish additional information on such problems as the prevalence of illegitimacy and incest, the size of family, the incidence of mongolism and twins, the average longevity, social status and conditions, size of head and brain, physical proportions and the stock from which they spring.

The material accumulated by the Trust under this head alone is so large and has necessarily occupied so much time in its collection that its analysis has not yet been begun, nor indeed can it be until the completion of the very considerable amount of work envisaged in the analysis of the 3,400 children already referred to. Some portion of it is, however, already proving of value for comparison with the normal data.

As regards the *third* and last of the three main lines of the work of the Trust—a macroscopic and microscopic examination of the brains of the normal and defective sections of the population—there had been collected up to October, 1937, the brains of 123 defectives and 82 normals, and this collection is as unique as it is invaluable. All the brains are prepared, preserved, and mounted in such a way as to make all of them available for present and future study by the present or future staffs, and this material is equally available for examination or research by any who care to apply for permission to do so. One observer (Professor S. E. Whitnall, of the University of Bristol) proposes to carry out an investigation on the calcarine and visual areas of normals and defectives. The present staff itself is engaged on an examination of the size and weight of brain, convolutional pattern, embryological errors, and pathological conditions of the defective brain as compared with the normal, whilst an intensive study of the histological cortical differences between defective and normal brains is already in operation.

From these several studies it is hoped and confidently expected that much light will be thrown on the basis and genesis of mental deficiency.

There is also now being prepared an illustrative atlas which will depict, photographically, the lateral and medial surfaces of the brains of a consecutive and entirely unselected series of 120 mental defectives, together with an account of their ages, family histories, mental reactions and clinical phenomena during life, for these data are known for all the cases. As each plate will also be accompanied by similar photographic illustrations of the normal brain of individuals of as nearly as possible the like chronological age, it is hoped to make the material available to a much wider circle of trained observers.

From this necessarily brief account of the activities of the Burden Trust and the work already accomplished it should be obvious that to complete the analysis of the wealth of human and pathological material already accumulated will require a far longer period of time than that originally visualized by the terms of the Trust. As regards the Bath scheme, this will be completed within the period originally planned, but useful extensions would be possible. One would be a thorough follow-up of certain remarkable families that have been discovered in the course of the investigation of the special groups.

If the work of the Trust is continued at Stoke Park Colony it is desirable that outside survey work should remain a feature of its activities. It is to be hoped that it may be possible to secure facilities in the city of Bristol. The importance of survey work in the area has recently been emphasized by the inauguration, by the University of Bristol, of a comprehensive Social Survey of the city, under the direction of Mr. Herbert Tout. During the past year Miss Griffiths has assisted in the work of the Child Guidance Clinic, the Trust having readily assented to a request by the city authorities that she should be allowed to undertake this work. At Stoke Park itself, of course, there will always be a wealth of material and opportunity for researches of all kinds on problems related to mental deficiency.

A special tribute must be paid to the generosity of Mrs. Burden. Her original gift, with the facilities available at Stoke Park Colony, made possible the comprehensively planned research here outlined, the results of which will be published in due course.

PUBLICATIONS OF THE TRUST.

R. G. GORDON, R. J. A. BERRY and R. M. NORMAN. " Neurological Abnormalities: their Occurrence and Significance as illustrated by an Examination of 500 Mental Defectives." *Journ. Neurol. and Psychopath.*, 1933, xiv., liv., 97.

R. J. A. BERRY. " The Problem of the Mental Defective." Reprinted from papers read at the Health Congress of the Royal Sanitary Institute held at Bristol in July, 1934.

R. J. A. BERRY and R. M. NORMAN. " Cerebral Structure and Mental Function as illustrated by a study of four defectives' brains." *Journ. Neurol. and Psychopath.*, 1934, xiv., lvi., 289.

R. J. A. BERRY. " Some of the Structural Abnormalities presented by the brains of thirty-one certified mental defectives." *Journ. Neurol. and Psychopath.*, 1935, xvi., lxi., 54.

R. M. NORMAN. " A Case of Juvenile Amaurotic Idiocy." *Journ. Neurol. and Psychopath.*, 1935, xv., lix., 219.

R. M. NORMAN. " Bilateral Atrophic Lobar Sclerosis following Thrombosis of the Superior Longitudinal Sinus." *Journ. Neurol. and Psychopath.* 1936, xvii., lxvi., 135.

J. A. FRASER ROBERTS, R. M. NORMAN and RUTH GRIFFITHS. " Studies on a Child Population: I. Definition of the Sample, Method of Ascertainment and Analysis of the Results of a Group Intelligence Test." *Annals of Eugenics*, 1935, vi., 319.

J. A. FRASER ROBERTS. " Heredity and Mental Deficiency." *British Medical Journal*, 1935, i., 413.

J. A. FRASER ROBERTS. " Twins." *Eugenics Review*, 1935, xxvii., 25.

R. J. A. BERRY. " Brain Size and Mentality." *British Medical Journal*, 1936, ii., 62.

R. J. A. BERRY. " What to do with the Mental Defective in Private Practice." *Medical Press and Circular*, 1936, cxciii., No. 5089.

J. A. FRASER ROBERTS and RUTH GRIFFITHS. " Studies on a Child Population: II. Re-tests on the Otis and Stanford-Binet Scales, with a Note on the Use of a Shortened Binet Scale." *Annals of Eugenics*, 1937, viii., 15.

J. A. FRASER ROBERTS, R. M. NORMAN and RUTH GRIFFITHS. " Studies on a Child Population: III. Intelligence and Family Size." *Annals of Eugenics*. In the press.

*In Preparation.*

J. A. FRASER ROBERTS, R. M. NORMAN and RUTH GRIFFITHS. " Studies on a Child Population: IV. On the Ascertainment of Low Stanford-Binet Intelligence Quotients."

R. M. NORMAN. " Some Observations on the Depth and Nerve Cell Content of the supra-granular layers of the Cerebral Cortex in Normal and Mentally Defective Persons."

R. J. A. BERRY. " An Atlas of Photographic Illustrations of 120 unselected Defective Brains, with details of the clinical, mental and neurological phenomena presented by each case during life, together with corresponding brains of a like series of normals."

J. A. FRASER ROBERTS. " Sex-linked microphthalmia sometimes associated with mental deficiency."

R. M. NORMAN. " An Example of Status Marmoratus of the Corpus Striatum and Cerebral Cortex."

